# A scalable, fully automated process for construction of sequence-ready barcoded libraries for 454

**DOI:** 10.1186/gb-2010-11-2-r15

**Published:** 2010-02-05

**Authors:** Niall J Lennon, Robert E Lintner, Scott Anderson, Pablo Alvarez, Andrew Barry, William Brockman, Riza Daza, Rachel L Erlich, Georgia Giannoukos, Lisa Green, Andrew Hollinger, Cindi A Hoover, David B Jaffe, Frank Juhn, Danielle McCarthy, Danielle Perrin, Karen Ponchner, Taryn L Powers, Kamran Rizzolo, Dana Robbins, Elizabeth Ryan, Carsten Russ, Todd Sparrow, John Stalker, Scott Steelman, Michael Weiand, Andrew Zimmer, Matthew R Henn, Chad Nusbaum, Robert Nicol

**Affiliations:** 1 Genome Sequencing Platform, Broad Institute of MIT and Harvard, 320 Charles St., Cambridge, MA 02141, USA; 2 Current address: Network Control Engineering, Akamai Technologies Inc., 8 Cambridge Center, Cambridge, MA 02142, USA; 3 Current address: Engineering, Google Inc., 5 Cambridge Center, Cambridge, MA 02142, USA; 4 Genome Sequencing and Analysis Program, Broad Institute of MIT & Harvard, 7 Cambridge Center, Cambridge, MA 02142, USA; 5 Current address: Genomic Technologies, Joint Genome Institute, Walnut Creek, CA 94598, USA

## Abstract

We present an automated, high throughput library construction process for 454 technology. Sample handling errors and cross-contamination are minimized via end-to-end barcoding of plasticware, along with molecular DNA barcoding of constructs. Automation-friendly magnetic bead-based size selection and cleanup steps have been devised, eliminating major bottlenecks and significant sources of error. Using this methodology, one technician can create 96 sequence-ready 454 libraries in 2 days, a dramatic improvement over the standard method.

## Abstract 2

An automated method for constructing libraries for 454 sequencing significantly reduces the cost and time required.

## Background

The emergence of next-generation sequencing technologies, such as the Roche/454 Genome Sequencer, the Illumina Genome Analyzer, the Applied Biosystems SOLiD sequencer and others, has provided the opportunity for both large genome centers and individual labs to generate DNA sequence data at an unprecedented scale [1]. However, as sequence output continues to increase dramatically, processes to generate sequence-ready libraries lag behind in scale. The minimum unit of sequence data (for example, lane or channel) already exceeds the amount required for small projects, such as viral or bacterial genomes, and will continue to increase. As a result, projects with large numbers of samples but small sequence per sample requirements become increasingly challenging to undertake in a cost-effective manner.

The 454 Genome Sequencer uses bead-in-emulsion amplification and a pyrosequencing chemistry to generate DNA sequence reads by synthesis [2]. Longer reads and shorter sequencing run times make the 454 platform a powerful tool for *de novo *assembly of small genomes, metagenomic profiling and amplicon sequencing compared with other next-generation sequencing platforms. However, these types of applications pose a challenge in that they require a relatively small number of reads from large numbers of samples. For example, for viruses such as HIV, the small (approximately 10 kb) genome size means that a single sample on even the smallest scale 454 picotiter plate configuration (1 region of a 16 region gasket) would yield over 1,500-fold coverage, vastly more coverage than required for genome assembly. Further, the standard 454 library construction protocol is not easily scalable and becomes a major cost driver relative to sequencing when modest numbers of reads are required from each sample. In addition, when sequencing large numbers of isolates of the same organism, the sequence identity between samples makes cross-contamination virtually impossible to detect without a molecular (sequence-based) tag. We set out to devise a laboratory process for high-throughput 454 sequencing that is able to generate large numbers of sequence-ready libraries at low cost per sample. Opportunities for sample mix-up errors or cross-contamination must be minimized and the process must also support efficient pooling of samples to avoid the cost of over-sequencing. Key requirements for this process include: plate-based processing of samples to enable handling by automation; redesign of process steps to be amenable to automation, particularly sample cleanup and size-selection steps; end-to-end barcoding, including barcoded input sample tubes and microtiter plates to support comprehensive sample tracking; molecular barcodes added to each DNA sample during library construction, which is read out as sequence, to support pooling before and sorting of reads after sequencing as well as easy identification of sample cross-contamination; automated construction of both fragment-read and paired (jumping) library types; low input DNA library construction; very limited human labor.

We have addressed each of these specifications in development of a high-throughput library construction process to support 454 sequencing. We were motivated by two key applications in particular, assembly of bacterial genomes and assembly and diversity analysis of small viral genomes, but the process is amenable to virtually any sequencing project with large numbers of samples.

## Results and discussion

### High-throughput library construction

We comprehensively redesigned the standard 454 library construction process for large-scale implementation of both fragment and 3-kb paired read library types. Table 1 describes the steps in the process, the scaling challenges of each step, the modifications that we have put in place for the high-throughput process and the benefits that each modification provides. This system utilizes a standard 96-well plate format and operates on the Velocity 11 Bravo, a small-footprint, liquid handling platform (see Additional files 1, 2 and 3 for process maps; a link to the Bravo automation protocol files can be found in Materials and methods), but can be implemented on many commercially available liquid handlers. The process is fully scalable and greatly decreases the potential for sample swaps and cross-contamination as well as operator-to-operator variability. We note that this process can also be carried out by hand (see Materials and methods).

**Table 1:** Improvements to library construction process

Process step	DNA fragmentation	Size selection/clean-ups	Adapter ligation	Multiplexing	Library quantification
Standard method	Nebulization	Column-based; agarose gel cuts	Un-tagged or one of 12 multiplex identifiers (MIDs) in tubes	Up to 12 samples pooled after library construction process	Ribogreen ssDNA assay
Drawback	Low throughput; Reduced yield	Not easily automated; opportunity for sample mix-up	Low throughput	Limited pool complexity	Limited accuracy and sensitivity
Modified method	Acoustic shear in 96-well plate	Solid phase reversible immobilization in 96-well plates	120 barcoded adapters in plate format	Up to 120 samples pooled after adapter ligation or enrichment step	qPCR
Benefit	Improved yield; increased throughput; automated setup	Amenable to automation; less opportunity for sample mix-up	Cross-contamination checks; high order multiplex within single region of PTP	Increased flexibility and pool complexity; decreased usage of LC reagents	Increased sensitivity; less input DNA required

LC, Library Construction; PTP, picotiter plate; qPCR, quantitative PCR; ssDNA, single-stranded DNA.

Samples are tracked end-to-end through the use of barcoded plasticware so that each step is captured in a laboratory information management system (LIMS). Since individual samples can come from many sources and sometimes in small batches, each sample enters the process in a two-dimensional barcoded microtube. The two-dimensional barcoded tubes (Thermo Matrix) are placed on the deck in racks of 96 where they are scanned for tracking in the LIMS. Samples are then transferred by the robot into 96-well plates labeled with standard code 128 barcodes for all downstream steps. Each sample also receives a unique, molecular barcode that is added at the adapter ligation step that allows for sample multiplexing and for downstream contamination checks (described below).

Implementation of automated library construction enables a single technician to produce 96 fragment libraries in 2 days or 24 3-kb jumping libraries in 3 days. This compares to an average throughput of six fragment libraries or four jumping libraries in the same time span using the standard method. The jumping library construction throughput has been kept lower to make cross-contamination even more unlikely, specifically because there are a large number of steps prior to adapter ligation and consequently more opportunity for sample cross-contamination. In this case, 24 samples in the 96-well plate are surrounded on all sides by either empty wells or an edge. The same sample layout scheme can be used for fragment library construction with smaller numbers of samples. Fragment library yield variation across a 96-well plate containing 24 samples is also shown in Additional file 4. See Additional file 5 for the layout of 24 samples in a 96-well plate.

### Reproducible, plate-based DNA shearing

The first step of the process is to shear DNA to a size range suitable for sequencing. Our goal was to implement a shearing method that would operate in a 96-well format with maximal yield of DNA fragments in the desired size range and with minimal process variability. Standard shearing methods using a nebulizer [3] are cumbersome, not well suited to high-throughput or automated genomic library construction and are prone to sample loss in tubes or vessels. Instead, we utilized the Covaris™ system for shearing, a method based on adaptive focused acoustic technology (see O'Brien [4] for an introduction). Adaptive focused acoustic technology has been successfully employed to fragment DNA for next-generation sequencing applications [5-8]. Compared with other methods, the Covaris system offers several major advantages for implementation in a high-throughput process. First, it is compatible with a 96-well format. Second, because it is performed in sealed wells with no contact between the device and the sample, cross-contamination is virtually eliminated and recovery of input volume is 100% (compared with as low as 50% using a nebulizer due to loss in the tubing and chamber). Third, the process is fully automated, so a full plate of samples can be sheared in a walk-away, pushbutton process. See Materials and methods for Covaris settings.

**Figure 1:**
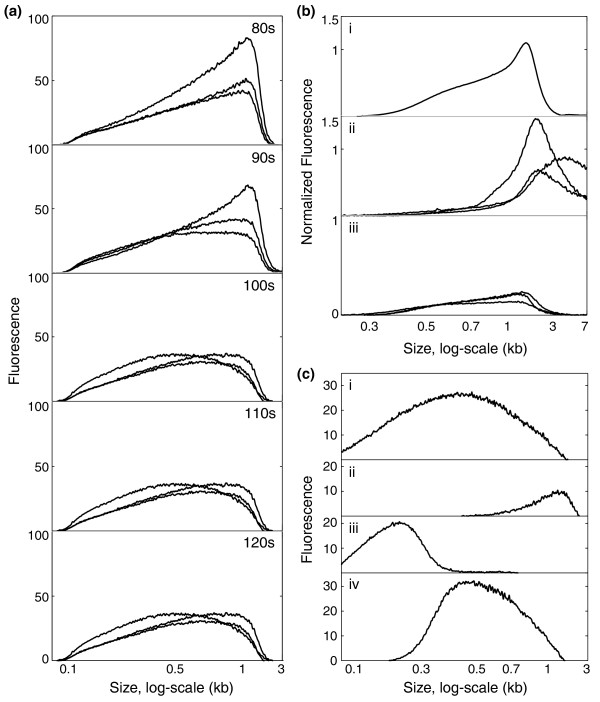
**Robust, optimized plate-based acoustic shearing of genomic DNA**. **(a) **Effect of time on shearing profile. Agilent Bioanalyzer traces of 3 μg human genomic DNA (Promega) diluted in 100 μl, aliquoted into an ABI PRISM™ Optical Reaction plate and sheared in the Covaris™ E210 under standard plate conditions (duty cycle = 5, intensity = 5, cycles per burst = 500) for increasing amounts of time (n = 3 for each timepoint). **(b) **Incomplete shears recovered by re-shearing. (i) Average shearing distribution (n = 27) of samples sheared for 100 seconds under standard conditions. (ii) An example of incomplete shearing seen in three attempts under standard conditions. (iii) Resultant fragment pattern after reshearing from (ii) with standard conditions. Each shear profile signal is plotted normalized to the maximum ladder fluorescence for the Bioanalyzer chip upon which the sample was run. **(c) **Dual high and low cutoff size-selection using para-magnetic beads (SPRI). Human genomic DNA (3 μg) was sheared under standard conditions, producing fragments ranging in size from less than 100 bp to approximately 4 kb (i). This shear product then underwent a 0.5× Solid Phase Reversible Immobilization (SPRI) reaction in which high molecular weight fragments were preferentially bound (ii). The supernatant was removed to a second tube and underwent a second 0.7× SPRI reaction where fragments below 300 bp were removed in the supernatant (iii). Fragments in the desired size range of 300 to 1,000 bp were eluted from the beads (iv).

The Covaris shearing process was extensively optimized for size range and yield in 96-well polypropylene plates using human genomic DNA (Figure 1a). We observed that duration of shearing has a predictable effect on the shear size profile. We have therefore used this as the primary variable in the optimization of shearing. Our current default conditions yield fragments ranging from 100 bp to over 1,000 bp but with a large proportion of the fragments in the 400- to 800-bp range, which is ideal for 454 FLX-Titanium read lengths (approximately 400 bases). Though nebulization can produce fragments in a tighter fragment length distribution, the above-described benefits of acoustic shearing make it an ideal method for a scalable process. Fragments outside the desired size range can be removed with subsequent size-selection steps (described below). Although we have observed a large fraction of fragments in the desired size range with the standard settings for >90% of genomic DNA samples (Figure 1b(i)), under-shearing is occasionally evident (Figure 1b(ii)), so it is important to assess the fragment size distribution (for example, with the Agilent BioAnalyzer). When the post-shear size distribution indicates incomplete shearing, samples can be re-sheared under standard conditions without apparent over-shearing, although some sample loss may be incurred (Figure 1b(iii)).

### Fully automated sample cleanup and size selection

Column-based reaction clean-ups and gel-based size selection steps are labor-intensive and resistant to automation. To make these processes scalable and amenable to automation, we redesigned these steps based on para-magnetic bead-based solid phase reversible immobilization (SPRI) of DNA. Binding of nucleic acids to carboxyl-para-magnetic microparticles can be made selective for molecular weight by manipulating concentrations of polyethylene glycol and salt to alter the ionic strength in solution [9]. Taking advantage of this, we use SPRI for three applications during library construction: as a buffer-exchange mechanism for washing in sample cleanup (without size selection) after fragment polishing and adapter ligation; as a low cutoff size selection to remove small (<300 bp) fragments after shearing; and as a high-and-low cutoff size selection, removing fragments outside the desired size range on both the low (<300 bp) and high (>1,000 bp) ends. We employ the latter method after library amplification in the 3-kb protocol and to remove fragments outside the desired size range from completed libraries (see Materials and methods for more details on SPRI).

For each application we have optimized the ratio of beads and buffer in the reaction. For buffer exchange, conditions include a higher bead to sample ratio, which ensures biding of nearly 100% of fragments. For low cutoff size selection, fragments >300 bp are bound to the beads and fragments <300 bp are removed in the supernatant. To perform accurate and scalable selection of DNA fragments in the desired size range (300 to 1,000 bases), a modified version of the low cutoff method is employed. First, fragments >1,000 bp are preferentially bound to beads and removed, and then the low cutoff size selection is applied as above. This provides a method to replace size selection by agarose gel that is accurate, scalable and amenable to automation (Figure 1c).

### Molecular barcoding

Molecular barcodes (also known as tags, indexes or multiplex identifiers) are short DNA sequences that appear at the ends (5' or 3') of every sequencing read, and function to link a read to its library source [10-14]. Read barcoding facilitates sample multiplexing [12-14] while increasing the ability to error-proof a sequencing process against cross-contamination events between libraries. The basic strategy for designing DNA barcodes has been to employ error correcting codes [5,14-17] and base selection filters (for example, limits to homopolymer length and terminal base restraints) that promote relatively short indices (<20 bases) with sufficient redundancy. Several effective barcoding schemes have been described (for example, [5,12-14,18]).

**Figure 2:**
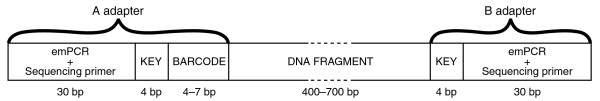
**Barcode adapter design**. Validated barcode sequences are added to the end of the 454 A adapter via DNA synthesis (Integrated DNA Technology). The lengths of each portion of the adapter and the approximate length of the insert are indicated. Validated barcodes are exactly 11 flows in length and range from 5 to 8 bases. emPCR, emulsion PCR.

To support efficient pooling of samples, we have incorporated molecular barcodes into the 454 library construction process by adding them to the 3' end of the 454 A adapter (Figure 2). To maximize the likelihood that identifiers can be called and compared accurately, the base sequences were defined using a linear ternary code [15] that is detected in ten nucleotide flows (the 454 nucleotide flow order is TACG). By exploiting the native format of 454 data, 'flow-space', this approach reduces the effects of hompolymer content on barcode sequence identification and trimming precision while striking a balance between keeping barcode sequences short to limit the fraction of total read bases lost to the barcode, and making them long enough to encode sufficient information content. The barcodes have a Hamming-distance [14-17] of three, meaning that three discrete sequencing errors must occur in the barcode portion of a read for it to be incorrectly identified as a separate, valid barcode.

Candidate barcode sequences were filtered to remove any with homopolymer runs longer than two bases and sequences starting with a G (the last base in the sequencing 'key') [12], giving a set of theoretical barcodes that passed the filtering step. A cytosine residue was added to the end of each barcode to separate it from the insert sequence, resulting in a set of barcodes that are exactly 11 flows long. 454 adapters bearing a subset of 144 filtered barcode sequences were synthesized and validated via representation in 454 shotgun libraries. In practice, we find that >97% of reads contain perfect barcodes. Therefore, though the design allows for it, in practice no additional error-correcting algorithms to recover miscalled barcodes has been implemented. We provide a full list of our validated barcodes as well as the ordering and annealing protocols in Additional file 1.

### Sample multiplexing

As discussed above, the increasing data yields of next-generation sequencers make it increasingly difficult to operate cost-efficiently on projects with large numbers of samples but small sequence-per-sample requirements. The standard 454 sequencing process allows for limited sample multiplexing; that is, running more than one sample at a time through physical separation of samples. Using a rubber gasket, the picotiter plate can be divided into 2, 4, 8 or 16 regions. This provides facile multiplexing but is inefficient, since as much as 50% of the picotiter plate is covered by the gasket, reducing the number of reads and thus increasing the cost per read. A much more efficient and flexible way to support sample multiplexing is to insert a molecular barcode sequence into each construct during library construction so that it can be read out in the sequence flowgram of each read. This not only enables straightforward multiplexing of any number of samples at any ratio, it also provides powerful quality control data, so that errors, mix-ups and contamination can be tracked to the level of the individual read.

Two molecular barcode-based multiplexing strategies have been validated using the in-house designed panel described above. The first approach, termed 'library pooling', provides a simple, accurate means of multiplexing for small-to-medium numbers of samples (for example, 20 to 40 libraries). In this method, plate-based library construction proceeds to completion as described above. Completed libraries are quantified using quantitative PCR (qPCR; see below), and then equal numbers of molecules from each library are pooled together. The pooled library molecules are then handled as a single sample through the emulsion PCR and sequencing processes. In this case the costs associated with emulsion PCR, breaking and enrichment of each library individually are reduced to the cost of processing a single tube through these steps.

The second approach, called 'adapted fragment pooling', is appropriate for projects with large numbers of samples that require relatively small numbers of reads. To maximally reduce costs, pooling should take place as early in the library construction process as possible. The earliest opportunity for pooling is immediately after adapter ligation. In this protocol up to 96 ligation reactions are pooled (10 μl each) into a single tube, which then proceeds through the final steps of library construction (immobilization, fill-in, and melt). One challenge with multiplexing at this stage arises from the presence of both active ligase and unincorporated adapters in the pool, which could result in the addition of a barcoded adapter to any unadapted fragments of a sample in the pool. To eliminate this possibility, we added a heat-inactivation step (10 minutes at 65°C) directly after barcoded-adapter ligation to eliminate ligase activity. Using this scheme we are able to pool samples immediately after ligation without any fragments being coupled to an incorrect barcode (see Additional file 1 for details of validation).

Both multiplexing strategies yield tight distributions of read representation across pooled samples, with 93% of barcodes returned within a two-fold spread of the mean sequence coverage. Using our automated, plate-based library construction process we have reduced the reagent cost per library from between 10-fold (non-mulitplexed) to 40-fold (multiplexed).

### Library quantification

Standard protocols for the quantification of 454 libraries (RiboGreen Assay, Life Technologies) cannot reliably detect library DNA concentrations below 0.1 ng/μl. Since only picogram amounts of material are required for the subsequent emulsion PCR, the implementation of a qPCR-based method to measure library concentration allows library construction from nanogram amounts of starting material [19,20] (see Meyer *et al*. [20] for a detailed protocol). For viral RT-PCR products, for example, we routinely perform production library construction from 100 to 200 ng of starting template per sample, and successful libraries have been made with as little as 1 ng.

## Conclusions

High-throughput DNA sequencing technologies from companies like Roche/454, Illumina, and ABI have made it possible to carry out large-scale sequencing projects such as the Thousand Genomes Project [21,22], The Cancer Genome Atlas [23], and other projects requiring many gigabases of sequence to reveal patterns in human-scale genomes. There are, however, many questions relevant to genomic aspects of human health and disease that can be answered without tens of millions of DNA sequence reads per sample, but rather where sequencing a large number of input samples is the key to biological discovery. Many projects require sequencing of many samples of very small genomes (for example, the Human Microbiome Project [24] or studies of viruses such as HIV and Dengue) or sequencing of large numbers of amplicons. For projects with modest sequence-per-sample requirements, technology development is required to support greater sample processing throughput and increased multiplexing to take best advantage of massively parallel sequencing technology. This report describes fully automated, highly scalable and cost-efficient methods for preparing sequence-ready libraries for the Roche/454 platform.

Substantial redesign of the sample preparation process was carried out to make it fully amenable to automation, a requirement for handling large numbers of samples. Some key innovations include: comprehensive barcoding - samples enter the process in individual two-dimensional barcoded microtubes, and all steps from sample entry to sequencing are tracked by barcoded plasticware, which virtually eliminates sample handling errors; (ii) DNA shearing is done in 96-well format - wells are sealed so that sample recovery is maximized; (iii) automated sample cleanup - columns have been replaced by bead-based liquid handling steps; (iv) automated size selection - agarose gels have been replaced by bead-based liquid handling steps. These last two steps were critical to removing manual steps and making the process compatible with automation. The full process has been implemented on a standard robotic liquid handling platform.

Molecular barcodes are incorporated into every sample, as an integral part of the library construction process. These are read out in the sequence reads, enabling facile creation and straightforward sorting of complex pools of samples for sequencing while at the same time providing a powerful and granular tool for quality assessment of the overall process. Our automated protocol is compatible with virtually all available barcoding schemes. For our process, we designed and validated (via successful synthesis, ligation, sequencing and sorting) a new set of error-correcting barcodes that are encoded in 454 flowspace.

In addition to scalability and barcoding, the automated process offers additional advantages. Process steps are standardized by automation, eliminating operator-introduced variability. A range of library types can be constructed, including approximately 400- to 800-bp fragments and approximately 3-kb 'jumping' constructs. Very little human labor is required, with the human labor component reduced by ten-fold or more, depending on library type. Finally, our approach is effective even with limiting amounts (<1 ng) of starting DNA.

As data yields from DNA sequencing platforms continue to grow, it becomes increasingly important to devise impedance-matched and cost-effective processes for preparation of sequence-ready libraries. This is particularly pressing for projects that call for sequencing of large numbers of samples each requiring a modest amount of data, such as small genomes or amplicons. We have addressed this need by developing sample preparation methods that are scalable, efficient and cost effective.

## Materials and methods

### Automated library construction protocols

Details of key plate configurations, labware definitions and aspirate/dispense conditions for the automated steps are available [25]. These files contain all the information required to operate our protocols on the Bravo platform, in the proprietary Velocity 11 format. In addition we have included the protocol for carrying out the plate-based library construction by hand, using a multi-channel pipette, for those without access to the liquid handling automation.

### Molecular barcode synthesis

All adapter oligonuceotides were ordered from Integrated DNA Technologies, (Coralville, IA, USA) with four phosphorothioate groups at both the 5' and 3' end to protect from nuclease digestion. Additionally, the B adapter contains a BioTEG group at the 5' end to facilitate adapted molecule immobilization in subsequent steps. All oligonucleotides were HPLC purified. The adapter oligo annealing and barcode validation methods are available in Additional file 1.

### Adaptive focused acoustic shearing of DNA

We use the Covaris E210 from Covaris Inc. (Woburn, MA, USA) and 96-well Optical Reaction Plates (ABI Cat. #4306737) for our plate-based shearing protocols. For automated transfers into and out of the unskirted optical reaction plate we used a standard 96-well PCR plate (Eppendorf Cat. # 951020401) as a holder into which the optical plate can sit and be defined on the deck of any automation.

Settings used for plate-based shearing of DNA are: Duty Cycle of 5; Intensity of 5; Cycle per Burst of 500; Seconds of 120; Well Plate of '96 well offset + 5 mm'.

It is important to avoid droplets being splashed and held at the top of the well during shearing as this will result in a population of unsheared fragments in the sample. To avoid this, we have found that use of optical stripcaps (ABI Cat. # 4323032) reduces the empty space inside the well and cuts down on splashes.

### Solid phase reversible immobilization

For low cutoff size selection we optimized the ratio of AMPure beads (Agencourt Biosciences, Beverly, MA, USA) and buffer to 0.7 times the volume of the DNA solution (that is, 70 ml beads added to 100 ml DNA) to remove fragments <300 bp. For buffer exchange, an excess of beads and buffer will ensure binding of nearly 100% of DNA fragments in solution. In our current production process we use 1.8 times the reaction volume or 1.8×; however, in practice values above 1× appear to be effective. For both of these implementations of SPRI, the DNA and bead solution are incubated for 5 minutes at room temperature. The magnetic beads with the DNA fragments reversibly bound to their surface are collected using a magnetic base station on the automation deck. Buffers and/or smaller fragments are removed with the supernatant. Beads are washed with 70% ethanol while still immobilized by the magnetic field. Ethanol is removed and the plate is moved from the magnet to another position on the deck to allow the beads to dry. Low ionic strength solution is added (10 mM Tris-Cl, pH 8.5) to dried beads to elute the DNA from the beads. DNA is then collected by returning the plate to its magnetic base and aspirating the eluate. Two different magnetic base stations are employed. In general, for wash steps in which DNA fraction remains on the beads, side magnets are used (DynaMag-96 Side; Invitrogen #123.31D) as they maximize the amount of supernatant that can be removed. For elution steps in which the DNA is removed in the supernatant, flat magnets are used (DynaMag-96 Bottom; Invitrogen #123.32D) as they maximally retain the beads. The exception is when reaction volumes are low (such as after fragment polishing), in which cases the bottom magnet is also used for washes.

A modified version of the low cutoff method is used to perform accurate and scalable selection of DNA fragments in the desired size range (300 to 1,000 bases). First, beads and buffer are added in a ratio (0.5 times the reaction volume) that promotes high-affinity binding of only large fragments. Fragments above 800 bp in size will preferentially remain bound to the bead fraction. The supernatant is then collected and added to a second reaction with beads and buffer at a higher ratio (0.7 times the reaction volume). From this mixture the eluate is collected as described above, removing fragments below the desired range (<300 bp) in the supernatant. This provides a method to replace size selection by agarose gel that is accurate, scalable and amenable to automation.

## Abbreviations

bp: base pair; LIMS: laboratory information management system; qPCR: quantitative PCR; SPRI: solid phase reversible immobilization.

## Authors' contributions

NJL managed much of the process development and drafted the manuscript, REL contributed significantly to the drafting of the manuscript, figure generation and data analysis and worked on the barcoding and qPCR, SA designed a lot of the automation scripts, PA participated in the molecular barcode design, AB oversaw the size-selection automation development, WB participated in the molecular barcode design, RD worked on the library construction development, RE worked on the validation and implementation of the molecular barcodes, GG worked on the validation of molecular barcodes and process development, LG worked on the validation of qPCR for library quantification, AH worked on post library construction multiplexing, CAH worked on the automation of the 3-kb jumping library process, DBJ oversaw the design of the molecular barcoding system, FJ worked on the integration of two-dimensional barcode scanning with the LIMS, DMcC worked on the library construction development, DP oversaw the sequencing and development process, KP managed a lot of the library construction development, TLP worked on the manual plate-based library construction process, KR worked on the shearing and barcoding processes, DR worked on the library construction development, ER worked on the barcode validation and the library construction development, CR managed the barcode implementation and study design, TS worked on the automation of library construction, JS worked on the integration of the lab processes with the LIMS, SS participated in the design and implementation of library construction improvements, MW worked on the optimization of shearing and library construction, AZ managed the integration of lab tracking into the LIMS, MRH participated in the low input and multiplexed library construction study design, CN guided and directed the application of the process improvements, and RN directed the design and implementation of the process improvements. All authors read and approved the final manuscript.

## Supplementary Material

Additional file 1 A Word document containing details and methods referred to but not described in the text. Click here for file

Additional file 2 A figure containing a process map for plate-based fragment library construction with details of automation used for each step. Click here for file

Additional file 3 A figure containing a process map for plate-based 3-kb jumping library construction with details of automation used for each step. Click here for file

Additional file 4 A figure illustrating variation in library yield across the plate. Click here for file

Additional file 5 A figure illustrating the layout of 24 samples in a 96-well plate. Click here for file

## References

[B1] Mardis ER Next-generation DNA sequencing methods. Annu Rev Genomics Hum Genet 2008 9 387 402 10.1146/annurev.genom.9.081307.164359 18576944

[B2] Margulies M Egholm M Altman WE Attiya S Bader JS Bemben LA Berka J Braverman MS Chen Y Chen Z Dewell SB Du L Fierro JM Gomes XV Godwin BC He W Helgesen S Ho CH Irzyk GP Jando SC Alenquer MLI Jarvie TP Jirage KB Kim J Knight JR Lanza JR Leamon JH Lefkowitz SM Lei M Genome sequencing in microfabricated high-density picolitre reactors. Nature 2005 437 376 380 16056220 10.1038/nature03959 PMC1464427

[B3] Bodenteich A Chissoe S Wang YF Roe BA Venter JC Shotgun cloning as the strategy of choice to generate templates for high-throughput dideoxynucleotide sequencing. Automated DNA Sequencing and Analysis Techniques 1993 London, UK: Academic Press 42 50

[B4] O'Brien WDJ Ultrasound-biophysics mechanisms. Prog Biophys Mol Biol 2007 93 212 255 16934858 10.1016/j.pbiomolbio.2006.07.010 PMC1995002

[B5] Quail MA Kozarewa I Smith F Scally A Stephens PJ Durbin R Swerdlow H Turner DJ A large genome center's improvements to the Illumina sequencing system. Nat Methods 2008 5 1005 1010 19034268 10.1038/nmeth.1270 PMC2610436

[B6] Wang X Sun Q McGrath SD Mardis ER Soloway PD Clark AG Transcriptome-wide identification of novel imprinted genes in neonatal mouse brain. PLoS ONE 2008 3 e3839 19052635 10.1371/journal.pone.0003839 PMC2585789

[B7] Kozarewa I Ning Z Quail MA Sanders MJ Berriman M Turner DJ Amplification-free Illumina sequencing-library preparation facilitates improved mapping and assembly of (G+C)-biased genomes. Nat Methods 2009 6 291 295 19287394 10.1038/nmeth.1311 PMC2664327

[B8] Yassour M Kaplan T Fraser HB Levin JZ Pfiffner J Adiconis X Schroth G Luo S Khrebtukova I Gnirke A Nusbaum C Thompson DA Friedman N Regev A *Ab initio *construction of a eukaryotic transcriptome by massively parallel mRNA sequencing. Proc Natl Acad Sci USA 2009 106 3264 3269 19208812 10.1073/pnas.0812841106 PMC2638735

[B9] Hawkins TL O'Connor-Morin T Roy A Santillan C DNA purification and isolation using a solid-phase. Nucleic Acids Res 1994 22 4543 4544 7971285 10.1093/nar/22.21.4543 PMC308491

[B10] Ooi SL Shoemaker DD Boeke JD A DNA microarray-based genetic screen for nonhomologous end-joining mutants in *Saccharomyces cerevisiae*. Science 2001 294 2552 2556 10.1126/science.1065672 11701889

[B11] Giaever G Chu AM Ni L Connelly C Riles L Véronneau S Dow S Lucau-Danila A Anderson K André B Arkin AP Astromoff A El-Bakkoury M Bangham R Benito R Brachat S Campanaro S Curtiss M Davis K Deutschbauer A Entian KD Flaherty P Foury F Garfinkel DJ Gerstein M Gotte D Güldener U Hegemann JH Hempel S Herman Z Functional profiling of the *Saccharomyces cerevisiae *genome. Nature 2002 418 387 391 10.1038/nature00935 12140549

[B12] Parameswaran P Jalili R Tao L Shokralla S Gharizadeh B Ronaghi M Fire AZ A pyrosequencing-tailored nucleotide barcode design unveils opportunities for large-scale sample multiplexing. Nucleic Acids Res 2007 35 e130 17932070 10.1093/nar/gkm760 PMC2095802

[B13] Binladen J Gilbert MT Bollback JP Panitz F Bendixen C Nielsen R Willerslev E The use of coded PCR primers enables high-throughput sequencing of multiple homolog amplification products by 454 parallel sequencing. PLoS ONE 2007 2 e197 17299583 10.1371/journal.pone.0000197 PMC1797623

[B14] Hamady M Walker JJ Harris JK Gold NJ Knight R Error-correcting barcoded primers for pyrosequencing hundreds of samples in multiplex. Nat Methods 2008 5 235 237 10.1038/nmeth.1184 18264105 PMC3439997

[B15] Ostergard PJR Upper bounds for q-ary covering codes. IEEE Trans Information Theory 1991 37 660 664 10.1109/18.79926

[B16] Hamming RW Error detecting and error correcting codes. Bell System Tech J 1950 29 147 160

[B17] He MX Petoukhov SV Ricci PE Genetic code, hamming distance and stochastic matrices. Bull Math Biol 2004 66 1405 1421 10.1016/j.bulm.2004.01.002 15294430

[B18] Frank DN BARCRAWL and BARTAB: software tools for the design and implementation of barcoded primers for highly multiplexed DNA sequencing. BMC Bioinformatics 2009 10 362 19874596 10.1186/1471-2105-10-362 PMC2777893

[B19] Meyer M Briggs AW Maricic T Höber B Höffner B Krause J Weihmann A Pääbo S Hofreiter M From micrograms to picograms: quantitative PCR reduces the material demands of high-throughput sequencing. Nucleic Acids Res 2008 36 e5 18084031 10.1093/nar/gkm1095 PMC2248761

[B20] Rutledge RG Stewart D A kinetic-based sigmoidal model for the polymerase chain reaction and its application to high-capacity absolute quantitative real-time PCR. BMC Biotechnol 2008 8 47 18466619 10.1186/1472-6750-8-47 PMC2397388

[B21] Kaiser J DNA sequencing: a plan to capture human diversity in 1000 genomes. Science 2008 319 395 395 10.1126/science.319.5862.395 18218868

[B22] Thousand Genomes Project http://www.1000genomes.org

[B23] McLendon R Friedman A Bigner D Van Meir EG Brat DJ Mastrogianakis GM Olson JJ Mikkelsen T Lehman N Aldape K Yung WK Bogler O Weinstein JN Berg S Vanden Berger M Prados M Muzny D Morgan M Scherer S Sabo A Nazareth L Lewis L Hall O Zhu Y Ren Y Alvi O Yao J Hawes A Jhangiani S Fowler G Comprehensive genomic characterization defines human glioblastoma genes and core pathways. Nature 2008 455 1061 1068 18772890 10.1038/nature07385 PMC2671642

[B24] NIH HMP Working Group Peterson J Garges S Giovanni M McInnes P Wang L Schloss JA Bonazzi V McEwen JE Wetterstrand KA Deal C Baker CC Di Francesco V Howcroft TK Karp RW Lunsford RD Wellington CR Belachew T Wright M Giblin C David H Mills M Salomon R Mullins C Akolkar B Begg L Davis C Grandison L Humble M Khalsa J Little AR The NIH Human Microbiome Project. Genome Res 2009 19 2317 2323 19819907 10.1101/gr.096651.109 PMC2792171

[B25] Automation and Plate-based Protocols http://www.broadinstitute.org/ftp/pub/papers/454barcodedlib/

