# Supplementary material for: A scalable, fully automated process for construction of sequence-ready barcoded libraries for 454
Source: Genome Biol. 2010 Feb 5;11(2):R15. doi: 10.1186/gb-2010-11-2-r15 (PMC2872875; doi:10.1186/gb-2010-11-2-r15)
Supplement: Additional file 1 — A Word document containing details and methods referred to but not described in the text. [file gb-2010-11-2-r15-S1.doc]

**A scalable, fully automated process for construction of sequence-ready barcoded libraries for 454**

Niall J. Lennon, Robert E. Lintner, Scott Anderson, Pablo Alvarez^*^, Andrew Barry, William Brockman^†^, Riza Daza, Rachel Erlich, Georgia Giannoukos*, Lisa Green, Andrew Hollinger, Cindi A. Hoover, David B. Jaffe*, Frank Juhn, Danielle McCarthy, Danielle Perrin, Karen Ponchner, Taryn Powers, Kamran Rizzolo, Dana Robbins, Elizabeth Ryan, Carsten Russ*, Todd Sparrow, John Stalker, Scott Steelman, Michael Weiand, Andrew Zimmer, Matthew R. Henn, Chad Nusbaum* and Robert Nicol^§^

Genome Sequencing Platform, Broad Institute of MIT & Harvard, 320 Charles St., Cambridge, MA 02141, USA

*Genome Sequencing and Analysis Program, Broad Institute of MIT & Harvard, 7 Cambridge Center, Cambridge, MA 02142, USA

^*^ Current address: Software Engineering, Akamai Technologies Inc., 8 Cambridge Center, Cambridge, MA 02142, USA

^†^ Current address: Software Engineering, Google Inc., 5 Cambridge Center, Cambridge, MA 02142, USA

**Additional file 1**

**Table of Contents**

1. Automated Library Construction Process Maps and Protocols
2. Validation of ligase-inactivation step
3. Broad-designed Molecular Barcode Information
4. Yield Variation in Plate-based Library Construction
5. Equipment used in Library Construction
6. Layout of 24 samples on a 96 well plate

1. Automated Library Construction Process Maps

Presented here are the process maps for both fragment (Additional file 2 - Figure S1) and 3kb (Additional file 3 - Figure S2) library construction protocols. The process steps are outlined, and required equipment and sample receptacle are indicated for each step.

2. Validation of Ligase-inactivation step

In the *adapted fragment pooling* protocol, large numbers of libraries are pooled immediately after the adapter ligation step. Each individual library in the pool is ligated to an adapter carrying a different barcode. Since adapters are still present in the pool, there is some concern that remaining ligase activity could join fragments to the wrong adapters after pooling. We added a heat inactivation step between ligation and pooling to overcome this. To validate this protocol, two barcode-ligated libraries were constructed, coupled to Mulitplex Identifiers (MIDs, Roche) 1 and 2 respectively. Ligations were carried out for 15 min at 25 º C, followed by heat inactivation for 10 minutes at 65 ºC. These samples were pooled together, along with mock ligation reactions containing MIDs 3 and 4, but no sample DNA. The mixture was put through standard ligation conditions (15 min at 25 º C) again. Library construction was completed and sequence generated. Sequence reads were binned by MID sequence detected, as shown in Table S1. Only reads containing MIDs #1 and #2 were detected, and all reads from the two genomes contained the appropriate corresponding MID. From this experiment we determined that the ligase was inactivated.

**Table S1: Barcode representation in ligase inactivation validation library**

|  | Total reads |
| --- | --- |
| MID#1 | 992 |
| MID#2 | 658 |
| MID#3 | 0 |
| MID#4 | 0 |

3. Broad-designed Molecular Barcode Information.

3.1 Table S2: **FLX Titanium Barcoded Adapter Sequences**

Key sequences are underlined. Barcode sequences are in **bold** font.

| **Adapter A Top Strand 5'-3'** | **Adapter A Bottom Strand 5'-3'** |
| --- | --- |
| CCATCTCATCCCTGCGTGTCTCCGACTCAG**CACGC** | GCGTGCTGAGTCGGAGA |
| CCATCTCATCCCTGCGTGTCTCCGACTCAG**CGCAAC** | GTTGCGCTGAGTCGGAGA |
| CCATCTCATCCCTGCGTGTCTCCGACTCAG**TGAAGC** | GCTTCACTGAGTCGGAGA |
| CCATCTCATCCCTGCGTGTCTCCGACTCAG**ACTTGC** | GCAAGTCTGAGTCGGAGA |
| CCATCTCATCCCTGCGTGTCTCCGACTCAG**TCACAC** | GTGTGACTGAGTCGGAGA |
| CCATCTCATCCCTGCGTGTCTCCGACTCAG**CGTGAC** | GTCACGCTGAGTCGGAGA |
| CCATCTCATCCCTGCGTGTCTCCGACTCAG**ACGCGC** | GCGCGTCTGAGTCGGAGA |
| CCATCTCATCCCTGCGTGTCTCCGACTCAG**CCTCTC** | GAGAGGCTGAGTCGGAGA |
| CCATCTCATCCCTGCGTGTCTCCGACTCAG**ACTCAC** | GTGAGTCTGAGTCGGAGA |
| CCATCTCATCCCTGCGTGTCTCCGACTCAG**AGACAC** | GTGTCTCTGAGTCGGAGA |
| CCATCTCATCCCTGCGTGTCTCCGACTCAG**CGACTC** | GAGTCGCTGAGTCGGAGA |
| CCATCTCATCCCTGCGTGTCTCCGACTCAG**AGCTTC** | GAAGCTCTGAGTCGGAGA |
| CCATCTCATCCCTGCGTGTCTCCGACTCAG**AAGCCGC** | GCGGCTTCTGAGTCGGAGA |
| CCATCTCATCCCTGCGTGTCTCCGACTCAG**CAAGAAC** | GTTCTTGCTGAGTCGGAGA |
| CCATCTCATCCCTGCGTGTCTCCGACTCAG**AGTTGGC** | GCCAACTCTGAGTCGGAGA |
| CCATCTCATCCCTGCGTGTCTCCGACTCAG**TATCAAC** | GTTGATACTGAGTCGGAGA |
| CCATCTCATCCCTGCGTGTCTCCGACTCAG**AGGCGGC** | GCCGCCTCTGAGTCGGAGA |
| CCATCTCATCCCTGCGTGTCTCCGACTCAG**CGGTATC** | GATACCGCTGAGTCGGAGA |
| CCATCTCATCCCTGCGTGTCTCCGACTCAG**TGACGAC** | GTCGTCACTGAGTCGGAGA |
| CCATCTCATCCCTGCGTGTCTCCGACTCAG**ACAAGGC** | GCCTTGTCTGAGTCGGAGA |
| CCATCTCATCCCTGCGTGTCTCCGACTCAG**AGACCTC** | GAGGTCTCTGAGTCGGAGA |
| CCATCTCATCCCTGCGTGTCTCCGACTCAG**ATACCAC** | GTGGTATCTGAGTCGGAGA |
| CCATCTCATCCCTGCGTGTCTCCGACTCAG**TCGCGGC** | GCCGCGACTGAGTCGGAGA |
| CCATCTCATCCCTGCGTGTCTCCGACTCAG**ATCTTAC** | GTAAGATCTGAGTCGGAGA |
| CCATCTCATCCCTGCGTGTCTCCGACTCAG**AACCAGC** | GCTGGTTCTGAGTCGGAGA |
| CCATCTCATCCCTGCGTGTCTCCGACTCAG**TTCGAGC** | GCTCGAACTGAGTCGGAGA |
| CCATCTCATCCCTGCGTGTCTCCGACTCAG**AAGGTGC** | GCACCTTCTGAGTCGGAGA |
| CCATCTCATCCCTGCGTGTCTCCGACTCAG**TCTTGGC** | GCCAAGACTGAGTCGGAGA |
| CCATCTCATCCCTGCGTGTCTCCGACTCAG**TAATCTC** | GAGATTACTGAGTCGGAGA |
| CCATCTCATCCCTGCGTGTCTCCGACTCAG**TCACCTC** | GAGGTGACTGAGTCGGAGA |
| CCATCTCATCCCTGCGTGTCTCCGACTCAG**TCCGCTC** | GAGCGGACTGAGTCGGAGA |
| CCATCTCATCCCTGCGTGTCTCCGACTCAG**TATTGAC** | GTCAATACTGAGTCGGAGA |
| CCATCTCATCCCTGCGTGTCTCCGACTCAG**AGTCGAC** | GTCGACTCTGAGTCGGAGA |
| CCATCTCATCCCTGCGTGTCTCCGACTCAG**ACGGCTC** | GAGCCGTCTGAGTCGGAGA |
| CCATCTCATCCCTGCGTGTCTCCGACTCAG**TGCGTTC** | GAACGCACTGAGTCGGAGA |
| CCATCTCATCCCTGCGTGTCTCCGACTCAG**TCTCGAC** | GTCGAGACTGAGTCGGAGA |
| CCATCTCATCCCTGCGTGTCTCCGACTCAG**CCAGGAC** | GTCCTGGCTGAGTCGGAGA |
| CCATCTCATCCCTGCGTGTCTCCGACTCAG**ACTCCTC** | GAGGAGTCTGAGTCGGAGA |
| CCATCTCATCCCTGCGTGTCTCCGACTCAG**TTCCTGC** | GCAGGAACTGAGTCGGAGA |
| CCATCTCATCCCTGCGTGTCTCCGACTCAG**TTCATAC** | GTATGAACTGAGTCGGAGA |
| CCATCTCATCCCTGCGTGTCTCCGACTCAG**CGTCGTC** | GACGACGCTGAGTCGGAGA |
| CCATCTCATCCCTGCGTGTCTCCGACTCAG**AAGGCAC** | GTGCCTTCTGAGTCGGAGA |
| CCATCTCATCCCTGCGTGTCTCCGACTCAG**AACAACTC** | GAGTTGTTCTGAGTCGGAGA |
| CCATCTCATCCCTGCGTGTCTCCGACTCAG**ACACGGAC** | GTCCGTGTCTGAGTCGGAGA |
| CCATCTCATCCCTGCGTGTCTCCGACTCAG**TGCCGAAC** | GTTCGGCACTGAGTCGGAGA |
| CCATCTCATCCCTGCGTGTCTCCGACTCAG**TATTCGTC** | GACGAATACTGAGTCGGAGA |
| CCATCTCATCCCTGCGTGTCTCCGACTCAG**TAGGAATC** | GATTCCTACTGAGTCGGAGA |
| CCATCTCATCCCTGCGTGTCTCCGACTCAG**CCGGCCAC** | GTGGCCGGCTGAGTCGGAGA |
| CCATCTCATCCCTGCGTGTCTCCGACTCAG**AATGGTAC** | GTACCATTCTGAGTCGGAGA |
| CCATCTCATCCCTGCGTGTCTCCGACTCAG**TCTCCGTC** | GACGGAGACTGAGTCGGAGA |
| CCATCTCATCCCTGCGTGTCTCCGACTCAG**AACCTGGC** | GCCAGGTTCTGAGTCGGAGA |
| CCATCTCATCCCTGCGTGTCTCCGACTCAG**ACGAAGTC** | GACTTCGTCTGAGTCGGAGA |
| CCATCTCATCCCTGCGTGTCTCCGACTCAG**TTCGTGGC** | GCCACGAACTGAGTCGGAGA |
| CCATCTCATCCCTGCGTGTCTCCGACTCAG**AACACAAC** | GTTGTGTTCTGAGTCGGAGA |
| CCATCTCATCCCTGCGTGTCTCCGACTCAG**TTCTTGAC** | GTCAAGAACTGAGTCGGAGA |
| CCATCTCATCCCTGCGTGTCTCCGACTCAG**TCCAAGTC** | GACTTGGACTGAGTCGGAGA |
| CCATCTCATCCCTGCGTGTCTCCGACTCAG**TTCGCGAC** | GTCGCGAACTGAGTCGGAGA |
| CCATCTCATCCCTGCGTGTCTCCGACTCAG**CCGGTCGC** | GCGACCGGCTGAGTCGGAGA |
| CCATCTCATCCCTGCGTGTCTCCGACTCAG**ACCTGAAC** | GTTCAGGTCTGAGTCGGAGA |
| CCATCTCATCCCTGCGTGTCTCCGACTCAG**AAGAGTTC** | GAACTCTTCTGAGTCGGAGA |
| CCATCTCATCCCTGCGTGTCTCCGACTCAG**TTGACAAC** | GTTGTCAACTGAGTCGGAGA |
| CCATCTCATCCCTGCGTGTCTCCGACTCAG**TCCAGAAC** | GTTCTGGACTGAGTCGGAGA |
| CCATCTCATCCCTGCGTGTCTCCGACTCAG**CGGTCTTC** | GAAGACCGCTGAGTCGGAGA |
| CCATCTCATCCCTGCGTGTCTCCGACTCAG**AAGGCCTC** | GAGGCCTTCTGAGTCGGAGA |
| CCATCTCATCCCTGCGTGTCTCCGACTCAG**ACTAATTC** | GAATTAGTCTGAGTCGGAGA |
| CCATCTCATCCCTGCGTGTCTCCGACTCAG**TGACCGTC** | GACGGTCACTGAGTCGGAGA |
| CCATCTCATCCCTGCGTGTCTCCGACTCAG**TGTCGGAC** | GTCCGACACTGAGTCGGAGA |
| CCATCTCATCCCTGCGTGTCTCCGACTCAG**AGGTTGTC** | GACAACCTCTGAGTCGGAGA |
| CCATCTCATCCCTGCGTGTCTCCGACTCAG**ACGAGAAC** | GTTCTCGTCTGAGTCGGAGA |
| CCATCTCATCCCTGCGTGTCTCCGACTCAG**TGGTGAAC** | GTTCACCACTGAGTCGGAGA |
| CCATCTCATCCCTGCGTGTCTCCGACTCAG**TCGTTGTC** | GACAACGACTGAGTCGGAGA |
| CCATCTCATCCCTGCGTGTCTCCGACTCAG**TTGTGTTC** | GAACACAACTGAGTCGGAGA |
| CCATCTCATCCCTGCGTGTCTCCGACTCAG**CCACGGTC** | GACCGTGGCTGAGTCGGAGA |
| CCATCTCATCCCTGCGTGTCTCCGACTCAG**TTGGAGGC** | GCCTCCAACTGAGTCGGAGA |
| CCATCTCATCCCTGCGTGTCTCCGACTCAG**TTATCGGC** | GCCGATAACTGAGTCGGAGA |
| CCATCTCATCCCTGCGTGTCTCCGACTCAG**AAGAAGAC** | GTCTTCTTCTGAGTCGGAGA |
| CCATCTCATCCCTGCGTGTCTCCGACTCAG**AACTGTTC** | GAACAGTTCTGAGTCGGAGA |
| CCATCTCATCCCTGCGTGTCTCCGACTCAG**TTCTCAAC** | GTTGAGAACTGAGTCGGAGA |
| CCATCTCATCCCTGCGTGTCTCCGACTCAG**CTTCCTTC** | GAAGGAAGCTGAGTCGGAGA |
| CCATCTCATCCCTGCGTGTCTCCGACTCAG**ATTCGTAC** | GTACGAATCTGAGTCGGAGA |
| CCATCTCATCCCTGCGTGTCTCCGACTCAG**CCTTCCGC** | GCGGAAGGCTGAGTCGGAGA |
| CCATCTCATCCCTGCGTGTCTCCGACTCAG**AGTCCGTC** | GACGGACTCTGAGTCGGAGA |
| CCATCTCATCCCTGCGTGTCTCCGACTCAG**TTGAACTC** | GAGTTCAACTGAGTCGGAGA |
| CCATCTCATCCCTGCGTGTCTCCGACTCAG**AACGAGGC** | GCCTCGTTCTGAGTCGGAGA |
| CCATCTCATCCCTGCGTGTCTCCGACTCAG**CCGTTCAC** | GTGAACGGCTGAGTCGGAGA |
| CCATCTCATCCCTGCGTGTCTCCGACTCAG**TCGAGGAAC** | GTTCCTCGACTGAGTCGGAGA |
| CCATCTCATCCCTGCGTGTCTCCGACTCAG**ACCGGAAGC** | GCTTCCGGTCTGAGTCGGAGA |
| CCATCTCATCCCTGCGTGTCTCCGACTCAG**ACGTTCCAC** | GTGGAACGTCTGAGTCGGAGA |
| CCATCTCATCCCTGCGTGTCTCCGACTCAG**AACGGAGTC** | GACTCCGTTCTGAGTCGGAGA |
| CCATCTCATCCCTGCGTGTCTCCGACTCAG**TTCGTTATC** | GATAACGAACTGAGTCGGAGA |
| CCATCTCATCCCTGCGTGTCTCCGACTCAG**ACCGTAATC** | GATTACGGTCTGAGTCGGAGA |
| CCATCTCATCCCTGCGTGTCTCCGACTCAG**ACCTTGGTC** | GACCAAGGTCTGAGTCGGAGA |
| CCATCTCATCCCTGCGTGTCTCCGACTCAG**TTAAGATTC** | GAATCTTAACTGAGTCGGAGA |
| CCATCTCATCCCTGCGTGTCTCCGACTCAG**TGGTTGGTC** | GACCAACCACTGAGTCGGAGA |
| CCATCTCATCCCTGCGTGTCTCCGACTCAG**TGTCCGGTC** | GACCGGACACTGAGTCGGAGA |
| CCATCTCATCCCTGCGTGTCTCCGACTCAG**AACCGTGTC** | GACACGGTTCTGAGTCGGAGA |
| CCATCTCATCCCTGCGTGTCTCCGACTCAG**TCGCGC** | GCGCGACTGAGTCGGAGA |
| CCATCTCATCCCTGCGTGTCTCCGACTCAG**CAACTAC** | GTAGTTGCTGAGTCGGAGA |
| CCATCTCATCCCTGCGTGTCTCCGACTCAG**CTGTAAC** | GTTACAGCTGAGTCGGAGA |
| CCATCTCATCCCTGCGTGTCTCCGACTCAG**CTTGTTC** | GAACAAGCTGAGTCGGAGA |
| CCATCTCATCCCTGCGTGTCTCCGACTCAG**CGCCTAC** | GTAGGCGCTGAGTCGGAGA |
| CCATCTCATCCCTGCGTGTCTCCGACTCAG**CGACGAC** | GTCGTCGCTGAGTCGGAGA |
| CCATCTCATCCCTGCGTGTCTCCGACTCAG**CGGATAC** | GTATCCGCTGAGTCGGAGA |
| CCATCTCATCCCTGCGTGTCTCCGACTCAG**CCTGGTC** | GACCAGGCTGAGTCGGAGA |
| CCATCTCATCCCTGCGTGTCTCCGACTCAG**ATCCGGC** | GCCGGATCTGAGTCGGAGA |
| CCATCTCATCCCTGCGTGTCTCCGACTCAG**ATACTTC** | GAAGTATCTGAGTCGGAGA |
| CCATCTCATCCCTGCGTGTCTCCGACTCAG**ATAAGTC** | GACTTATCTGAGTCGGAGA |
| CCATCTCATCCCTGCGTGTCTCCGACTCAG**ATTACAC** | GTGTAATCTGAGTCGGAGA |
| CCATCTCATCCCTGCGTGTCTCCGACTCAG**AGCGAAC** | GTTCGCTCTGAGTCGGAGA |
| CCATCTCATCCCTGCGTGTCTCCGACTCAG**AGTCGTC** | GACGACTCTGAGTCGGAGA |
| CCATCTCATCCCTGCGTGTCTCCGACTCAG**AGTAGGC** | GCCTACTCTGAGTCGGAGA |
| CCATCTCATCCCTGCGTGTCTCCGACTCAG**ACACGTC** | GACGTGTCTGAGTCGGAGA |
| CCATCTCATCCCTGCGTGTCTCCGACTCAG**ACTCCAC** | GTGGAGTCTGAGTCGGAGA |
| CCATCTCATCCCTGCGTGTCTCCGACTCAG**ACGCGGC** | GCCGCGTCTGAGTCGGAGA |
| CCATCTCATCCCTGCGTGTCTCCGACTCAG**ACGATTC** | GAATCGTCTGAGTCGGAGA |
| CCATCTCATCCCTGCGTGTCTCCGACTCAG**ACCGCAC** | GTGCGGTCTGAGTCGGAGA |
| CCATCTCATCCCTGCGTGTCTCCGACTCAG**AATTCGC** | GCGAATTCTGAGTCGGAGA |
| CCATCTCATCCCTGCGTGTCTCCGACTCAG**AACTATC** | GATAGTTCTGAGTCGGAGA |
| CCATCTCATCCCTGCGTGTCTCCGACTCAG**AACGTGC** | GCACGTTCTGAGTCGGAGA |
| CCATCTCATCCCTGCGTGTCTCCGACTCAG**TGCCTTC** | GAAGGCACTGAGTCGGAGA |
| CCATCTCATCCCTGCGTGTCTCCGACTCAG**TGACGTC** | GACGTCACTGAGTCGGAGA |
|  |  |
| **Adapter B Top Strand 5'-3'** | **Adapter B Bottom Strand 5'-3'** |
| CCTATCCCCTGTGTGCCTTGGCAGTCTCAG | CTGAGACTGCCA |

Modifications: All oligos get four phosphorothioate groups at both the 5’ and 3’ end to protect from nuclease digestion. Additionally the B adapter is ordered with a BioTEG group at the 5’ end. All oligonucleotides were HPLC purified.

3.2 **Adapter Annealing Method**

*Perform steps 1-4 separately for adapters A and B:*

1. Dilute Top and Bottom Strands to 800 pmoles/ul each in 1X STE, and place on shaker to resuspend, vortexing occasionally, for 30 min. *i.e.* if 50 nmoles total, add 62.5ul STE STE Buffer = 10 mM Tris pH 8.0, 50 mM NaCl, 1 mM EDTA
2. Mix Top and Bottom Strands in 1:1 molar ratio
3. If adapters are in 0.2 mL tubes or PCR plate, use thermocycler to anneal:

95ºC for 1 minute

Ramp to 15ºC at 0.1ºC/second

14º C forever

**OR**

1. If adapters are in 1.5mL eppendorf tubes, use water bath or heat block:

95ºC 4 minutes

Turn off water bath/heat block and allow to cool slowly to room temperature.

1. Mix cooled (or thawed, if previously frozen) A and B adapters in 1:1 molar ratio

(*i.e.* Final concentration of each adapter is 200 picomoles/ul. Before final use, adapter plates are diluted in half with TE buffer and 2 ul of this solution is added to each samples.)

1. Store adapters at -20ºC until use.

3.3 **Barcoded Adapter Validation Method**

To QC the oligonucleotides ordered from an external vendor the following method was devised.

1. 1ul of each of the annealed barcoded adapter mix (A and B adapters) is removed to a 1.5ml eppendorf tube.
2. This pool of 96 (or however many oligos were in the order) is then used as the adapter mix for ligation to a control DNA during library construction. Note: The control DNA can be any good quality sample, preferably one that has been sequenced previously or is an otherwise known material.
3. The library made with the adapter pool is sequenced as normal on the 454 machine. Reads are separated post-sequencing by binning of the known, expected barcode sequences.
4. A barcoded adapter oligo is deemed to pass validation if it is present in the final pool at a frequency plus or minus 1.5 standard deviations of the mean read representation for all barcodes.

4. Yield Variation in Plate-based Library Construction

ssDNA yields from the automated, plate-based library construction process vary according to the type and amounts of starting materials used. Additional file 4 (Figure S3) illustrates the typical yields from 24 microbial genomic libraries where the starting material in each well was 3ug of DNA. In this case the solid line indicates the average yield and the shaded area shows the variation across all 24 samples. The Coefficient of Variation for the yield of library fragment at 700bp for this set was 0.61. We have set an arbitrary library yield QC of 5 Fluorescence Units on the Bioanalyzer. Using this QC metric, the fail rate for library QC in a single plate has been between 1-4%. It should be noted that the average Fluorescence Units for samples failing the QC was 4.8, or an average ssDNA concentration of 83.4 ng/ul. This translates to ~1.27 x 10^11^ copies/ul and is still adequate material from which to make the standard library dilutions (1 x 10^8^, 2 x10^6^ and 2 x 10^5^ copies/ul) prior to emulsion PCR.

Variation in yields from amplicon libraries is difficult to assay directly as these fragments are pooled by volume immediately after adapter ligation. A proxy for direct measurement may be the relative representation of amplicons in the final read counts (although this representation may also be altered by differing performance in emulsion PCR). In this case we have seen sample read representation vary by about 2-fold from highest to lowest number of reads. The dropout rate for pooled amplicons is also 1-4% depending on the library.

5. Equipment used in Library Construction

The two main pieces of equipment required for the automated construction of 454 libraries in 96 well plates, as laid out in this manuscript are:

1. Covaris E210 (Covaris Inc, Woburn, MA). Approximate retail price: $113,000.

2. Bravo Automated Liquid Handling Platform (Agilent Product Number G5409A) with the 96LT Disposable Tip pipette head (Option number 178). Both from Agilent Technologies (Santa Clara, CA). Approximate retail price of this configuration: $90,000.

6. Layout of 24 samples on a 96 well plate

Additional file 5 (Figure S4) illustrates the plate layout for automated library construction when only 24 samples are processed concurrently.

**Supplementary Information Figure Legends**

**Figure S1. Fragment Library Construction Process Map**

All major steps in the LC process are shown here with the sample receptacle (parentheses) and automation/equipment indicated. First and last boxes represent the inputs and outputs of this process, respectively. Inputs are DNA samples arrayed and associated with 2D barcoded tubes. Outputs are pre-QC single stranded DNA libraries that are transferred into 2D barcoded tubes, the tube barcodes are then scanned associating the final library with the input material in the laboratory informatics management system database (LIMS).

**Figure S2. 3kb Paired-end Library Construction Process Map**

First and last boxes represent the inputs and outputs of this process, respectively. Inputs to this process are DNA samples that have been individually sheared to ~3kb using a hydroshear apparatus.

**Figure S3. Library Construction Yield Variation**

Variation in yield across 24 genomic DNA samples that have gone through plate-based library construction is shown. Yield was assessed by Agilent BioAnalyzer analysis of single-stranded RNA library concentration and size distribution. Solid line represents mean yield and shaded area the variation across all 24 samples on the plate.

**Figure S4. Layout of 24 samples on a 96 well plate**

The shaded circles represent the sample-containing wells. Each sample is surrounded by either an edge or an empty well (non-shaded circles).
