# Supplementary material for: A scalable, fully automated process for construction of sequence-ready barcoded libraries for 454
Source: Genome Biol. 2010 Feb 5;11(2):R15. doi: 10.1186/gb-2010-11-2-r15 (PMC2872875; doi:10.1186/gb-2010-11-2-r15)
Supplement: Additional file 2 — A figure containing a process map for plate-based fragment library construction with details of automation used for each step. [file gb-2010-11-2-r15-S2.ppt]

## Slide 1
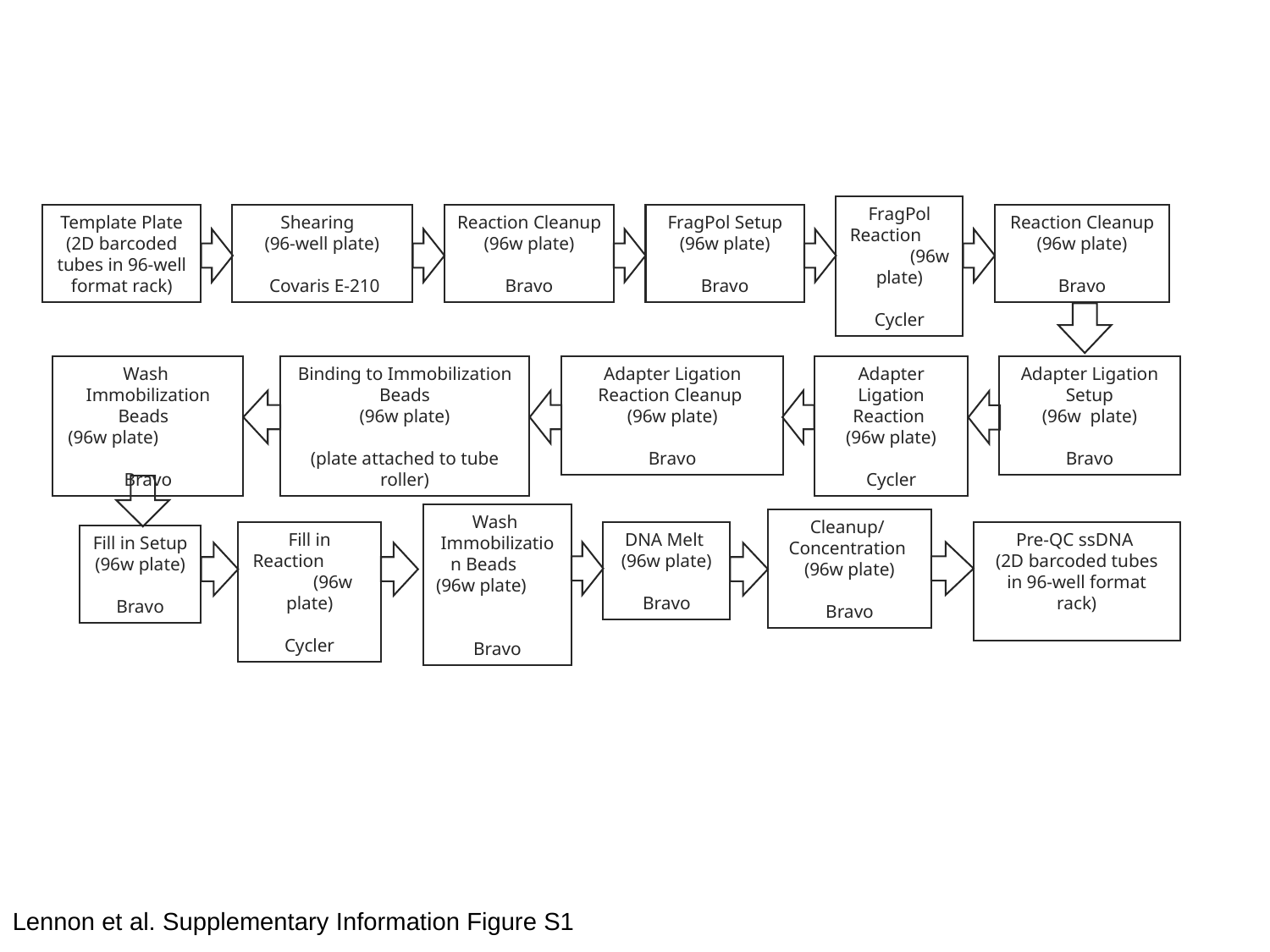

FragPol Reaction (96w plate)
Cycler
Template Plate
(2D barcoded tubes in 96-well format rack)
Shearing
(96-well plate)
 Covaris E-210
Reaction Cleanup
(96w plate)
Bravo
FragPol Setup
(96w plate)
Bravo
Reaction Cleanup (96w plate)
Bravo
Wash
Immobilization Beads
(96w plate)
Bravo
Binding to Immobilization Beads
(96w plate)
(plate attached to tube roller)
Adapter Ligation Reaction Cleanup
(96w plate)
Bravo
Adapter Ligation Reaction
(96w plate)
Cycler
Adapter Ligation Setup
(96w plate)
Bravo
Wash
Immobilization Beads
(96w plate)
Bravo
Cleanup/ Concentration
(96w plate)
Bravo
Fill in Reaction (96w plate)
Cycler
DNA Melt
(96w plate)
Bravo
Pre-QC ssDNA
(2D barcoded tubes in 96-well format rack)
Fill in Setup
(96w plate)
Bravo
Lennon et al. Supplementary Information Figure S1
