# Supplementary material for: A scalable, fully automated process for construction of sequence-ready barcoded libraries for 454
Source: Genome Biol. 2010 Feb 5;11(2):R15. doi: 10.1186/gb-2010-11-2-r15 (PMC2872875; doi:10.1186/gb-2010-11-2-r15)
Supplement: Additional file 3 — A figure containing a process map for plate-based 3-kb jumping library construction with details of automation used for each step. [file gb-2010-11-2-r15-S3.ppt]

## Slide 1
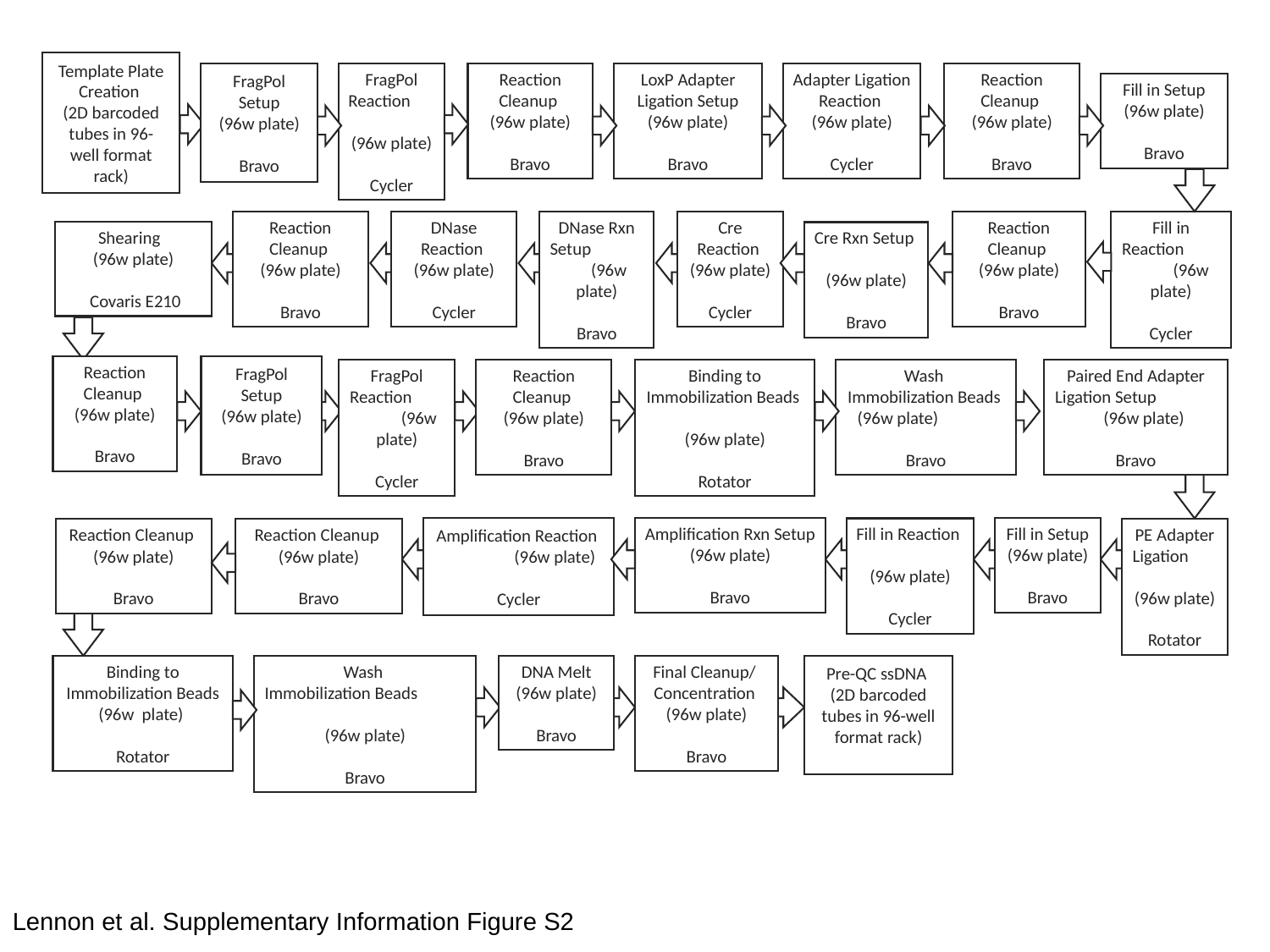

Template Plate Creation
(2D barcoded tubes in 96-well format rack)
FragPol Setup
(96w plate)
Bravo
FragPol Reaction (96w plate)
Cycler
Reaction Cleanup
(96w plate)
Bravo
LoxP Adapter Ligation Setup
(96w plate)
Bravo
Adapter Ligation Reaction
(96w plate)
Cycler
Reaction Cleanup
(96w plate)
Bravo
Fill in Setup
(96w plate)
Bravo
Reaction Cleanup
(96w plate)
Bravo
DNase Reaction
(96w plate)
Cycler
DNase Rxn Setup (96w plate)
Bravo
Cre Reaction
(96w plate)
Cycler
Reaction Cleanup
(96w plate)
Bravo
Fill in Reaction (96w plate)
Cycler
Shearing
(96w plate)
 Covaris E210
Cre Rxn Setup (96w plate)
Bravo
Reaction Cleanup
(96w plate)
Bravo
FragPol Setup
(96w plate)
Bravo
FragPol Reaction (96w plate)
Cycler
Reaction Cleanup
(96w plate)
Bravo
Binding to Immobilization Beads
(96w plate)
Rotator
Wash
Immobilization Beads
(96w plate)
Bravo
Paired End Adapter Ligation Setup (96w plate)
Bravo
Amplification Reaction (96w plate)
Cycler
Amplification Rxn Setup
(96w plate)
Bravo
Fill in Reaction (96w plate)
Cycler
Fill in Setup
(96w plate)
Bravo
PE Adapter Ligation
(96w plate)
Rotator
Reaction Cleanup
(96w plate)
Bravo
Reaction Cleanup
(96w plate)
Bravo
Binding to Immobilization Beads
(96w plate)
Rotator
Wash
Immobilization Beads
(96w plate)
Bravo
DNA Melt
(96w plate)
Bravo
Final Cleanup/ Concentration
(96w plate)
Bravo
Pre-QC ssDNA
(2D barcoded tubes in 96-well format rack)
Lennon et al. Supplementary Information Figure S2
