# Supplementary figures and images for: A scalable, fully automated process for construction of sequence-ready barcoded libraries for 454
Source: Genome Biol. 2010 Feb 5;11(2):R15. doi: 10.1186/gb-2010-11-2-r15 (PMC2872875; doi:10.1186/gb-2010-11-2-r15)

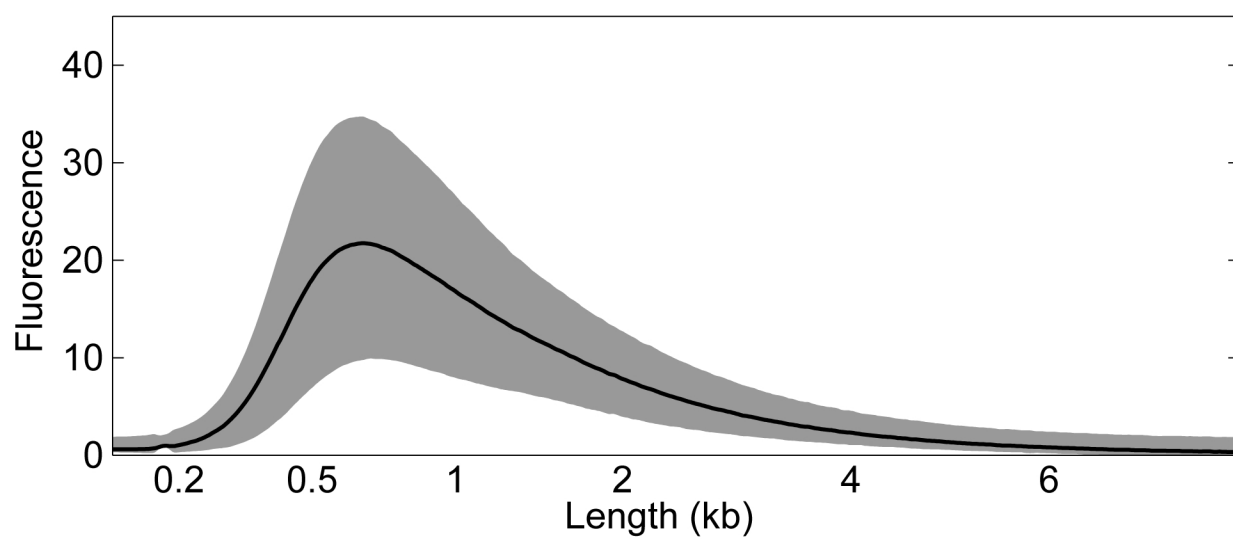

Supplement: Additional file 4 — A figure illustrating variation in library yield across the plate. [file gb-2010-11-2-r15-S4.pdf]

## Slide 1
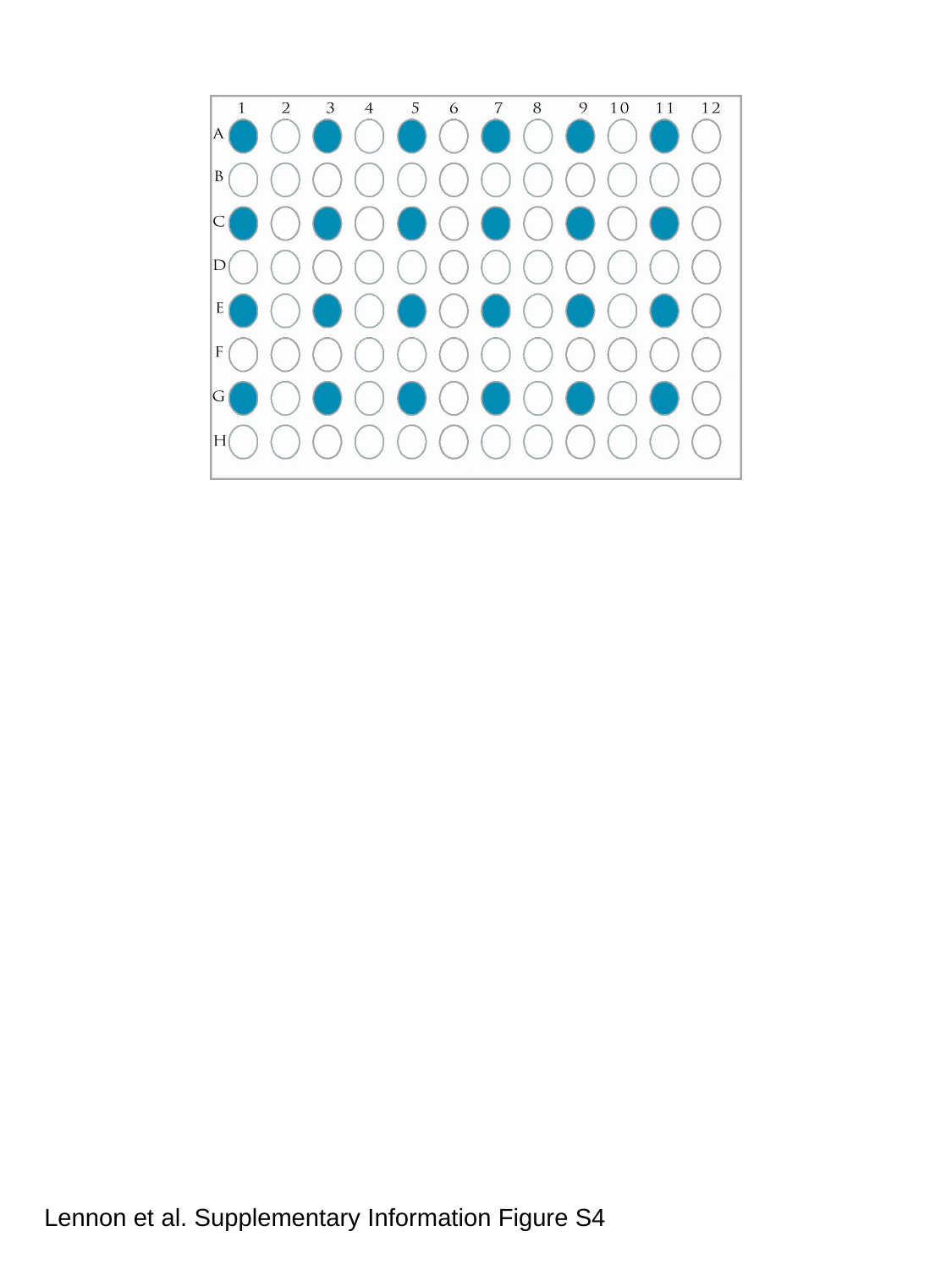

Lennon et al. Supplementary Information Figure S4

Supplement: Additional file 5 — A figure illustrating the layout of 24 samples in a 96-well plate. [file gb-2010-11-2-r15-S5.pptx]
